# 1-[4-(Di­methyl­amino)­benzyl­idene]-4-*o*-tolyl­thio­semicarbazide

**DOI:** 10.1107/S1600536813012890

**Published:** 2013-05-18

**Authors:** Rui-Yun Huang, Qin-Juan Xu, Li-Rong Lin

**Affiliations:** a Department of Chemistry, Xiamen University, Xiamen 361005, People’s Republic of China

## Abstract

The asymmetric unit of the title compound, C_17_H_20_N_4_S, contains two independent mol­ecules, the main difference between them being the dihedral angles between the benzene rings [19.99 (17) and 9.72 (17)°]. The mol­ecules both have a *trans* conformation about the C=N double bond and intra­molecular C—H⋯S and N—H⋯N hydrogen bonds are observed in both mol­ecules. In the crystal, mol­ecules are linked by weak N—H⋯S hydrogen bonds with graph-set motif *R*_2_^2^(8). In each mol­ecule, all but one of the N atoms and both the S atoms are involved in hydrogen bonding.

## Related literature

For details of anion recognition, see: Sessler *et al.* (2006 ▶); Amendola *et al.* (2006 ▶); Fahlbusch *et al.* (2006 ▶); Gale & Quesada (2006 ▶); Perez & Riera (2008 ▶); Willans *et al.* (2009 ▶); Amendola & Fabbrizzi (2009 ▶); Haridas *et al.* (2012 ▶). For applications of thio­semicarbazides, see: Basuli *et al.* (1998 ▶); Pandeya *et al.* (1999 ▶); Kowol *et al.* (2010 ▶). For thio­semicarbazones acting as anion acceptors, see: Chikate *et al.* (2005 ▶); Krisitin (2005 ▶). For details of the synthesis of the Schiff base ligand, see: Pouralimardan *et al.* (2007 ▶). For hydrogen-bond motifs, see: Bernstein *et al.* (1995 ▶).
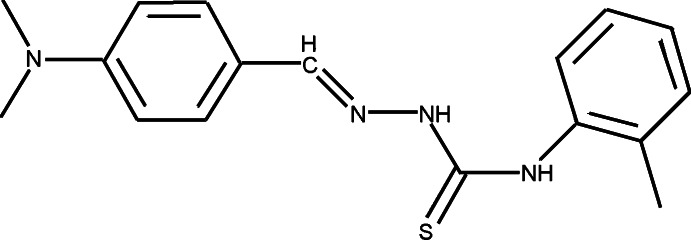


## Experimental

### 

#### Crystal data

C_17_H_20_N_4_S
*M* *_r_* = 312.43
Triclinic, 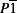

*a* = 9.172 (3) Å
*b* = 12.224 (4) Å
*c* = 15.563 (5) Å
α = 86.933 (6)°
β = 86.990 (7)°
γ = 70.382 (6)°
*V* = 1640.3 (9) Å^3^
*Z* = 4
Mo *K*α radiation
μ = 0.20 mm^−1^
*T* = 173 K
0.20 × 0.15 × 0.03 mm

#### Data collection

Brucker SMART APEX diffractometer
Absorption correction: multi-scan (*SADABS*; Sheldrick,2008 ▶) *T*_min_ = 0.961, *T*_max_ = 0.994
7929 measured reflections
5640 independent reflections
3127 reflections with *I* > 2σ(*I*)
*R*_int_ = 0.027

#### Refinement

*R*[*F* ^2^ > 2σ(*F* ^2^)] = 0.058
*wR*(*F* ^2^) = 0.155
*S* = 0.95
5640 reflections
419 parameters
H atoms treated by a mixture of independent and constrained refinement
Δρ_max_ = 0.29 e Å^−3^
Δρ_min_ = −0.17 e Å^−3^

### 

Data collection: *SMART* (Bruker, 2001 ▶); cell refinement: *SAINT* (Bruker, 2001 ▶); data reduction: *SAINT*; program(s) used to solve structure: *SHELXS97* (Sheldrick, 2008 ▶); program(s) used to refine structure: *SHELXL97* (Sheldrick, 2008 ▶); molecular graphics: *ORTEP-3 for Windows* (Farrugia, 2012 ▶); software used to prepare material for publication: *SHELXL97*.

## Supplementary Material

Crystal structure: contains datablock(s) I, global. DOI: 10.1107/S1600536813012890/bx2440sup1.cif

Structure factors: contains datablock(s) I. DOI: 10.1107/S1600536813012890/bx2440Isup2.hkl

Supplementary material file. DOI: 10.1107/S1600536813012890/bx2440Isup3.mol

Supplementary material file. DOI: 10.1107/S1600536813012890/bx2440Isup4.cml

Additional supplementary materials:  crystallographic information; 3D view; checkCIF report

## Figures and Tables

**Table 1:** Hydrogen-bond geometry (Å, °)

*D*—H⋯*A*	*D*—H	H⋯*A*	*D*⋯*A*	*D*—H⋯*A*
N4—H4*N*⋯N2	0.95 (3)	2.05 (3)	2.587 (3)	114 (3)
C12—H12*A*⋯S1	0.93	2.64	3.239 (4)	123
C29—H29*A*⋯S2	0.93	2.57	3.257 (4)	131
N7—H7*N*⋯S1^i^	0.86	2.61	3.458 (3)	170
N3—H3*N*⋯S2^ii^	0.86	2.59	3.439 (3)	171
N8—H8*N*⋯N6	0.97 (3)	2.01 (3)	2.587 (4)	116 (2)

Symmetry codes: (i) 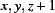; (ii) 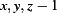.

**Fig. 1.**
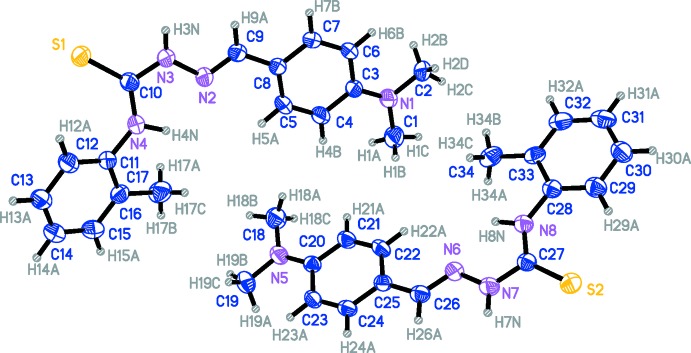
ORTEP plot of the title compound. The thermal ellipsoids are drawn at 30% probability level.

## supplementary crystallographic information

### Comment

Recently, anions recognition and sensing has attracted growing attentions due to their important roles in the fields of environmental science, biology, catalysis and medicine (Sessler *et al.* 2006). Substantial progress has been made in the development of anion receptors (Amendola *et al.* 2006, Gale *et al.* 2006, Perez & Riera 2008, Amendola *et al.* 2009, Kowol *et al.* 2010, Haridas *et al.* 2012). Thiosemicarbazide has been widely used in catalysis, drug, bacterialcide, flotation agent, (Pouralimardan *et al.* 2007). Some thiosemicarbazide derivatives show interesting biological effects, such as anticancer and anti-HIV properties (Pandeya *et al.* 1999; Fahlbusch *et al.* 2006). There are also a few reports of thiosemicarbazide acting as anion acceptors (Chikate *et al.* 2005). The introduction of a strong electron-donating group such as the *N*,*N*-dimethylanilino group through a double bond imparts new properties that are not commonly observed for the parent thiourea. As part of our work in this area, we report here the synthesis and structure of the title compound.The asymmetric unit of the title compound, C_17_H_20_N_4_S, consists of two crystallographically independent molecules. The main differences of both molecules are the dihedral angles between the benzene rings, 19.99 (17)° and 9.72 (17)°. In the crystal, molecules are linked by weak intermolecular N—H···S hydrogen bonds with set graph-motif R^2^_2_(8) (Bernstein *et al.*, 1995). Intramolecular C—H···S and N—H···N hydrogen bonds are observe. In both molecules, all of the N atoms and two of the S atoms are involved in hydrogen bonding, with an average H···S distance of 2.61Å and N— H···S angles in the range 170–171°. The molecules have a *trans* configuration about the C=N double bond.

### Experimental

The title compound was synthesized by refluxing an ethanol solution (20 ml) of 4-(dimethylamino) benzaldehyde (10 mmol) and 4-(4-methylphenyl)-3-thiosemicarbazide (10 mmol) for 3 h. The resulting yellow and clear solution was then cooled to room temperature. Light-yellow crystals were obtained, filtered and washed with cold ethanol. Crystals suitable for X-ray diffraction analysis were obtained by slow evaporation of an acetonitrile and dichromethane mixed solution. M.P. 179~180 °C, MS (APCI) m/*z* (%): *M*^+^ 311.4, ^1^H-NMR (CDCl_3_), δ (p.p.m.): 9.53 (s,NH), 9.00 (s, NH), 7.84 (s, CH=N), 7.77 (dd, 1H, *J* = 4 Hz, ArH), 7.63 (d, 2H, *J* = 12 Hz, ArH), 7.32~7.30 (m, 1H, ArH), 7.25~7.23 (m, 2H, ArH), 7.02~6.99 (m, 2H, ArH), 3.10 (s, 6H, CH_3_), 2.39 (s, 3H, CH_3_).

### Refinement

Treatment of hydrogen atoms in the least-squares refinement: some constrained, some independent. For constrained, H atoms were positioned geometrically (C—H = 0.95–0.98 A ° and N—H = 0.88 A °) and refined as riding, with *U*_iso_(H) = 1.2Ueq(carrier) or 1.5Ueq(methyl C).

### Crystal data

**Table:**

C_17_H_20_N_4_S	*Z* = 4
*M**_r_* = 312.43	*F*(000) = 664
Triclinic, *P*1	*D*_x_ = 1.265 Mg m^−^^3^
Hall symbol: -P 1	Melting point: 452 K
*a* = 9.172 (3) Å	Mo *K*α radiation, λ = 0.71073 Å
*b* = 12.224 (4) Å	Cell parameters from 9709 reflections
*c* = 15.563 (5) Å	θ = 1.8–25.0°
α = 86.933 (6)°	µ = 0.20 mm^−^^1^
β = 86.990 (7)°	*T* = 173 K
γ = 70.382 (6)°	Block, yellow
*V* = 1640.3 (9) Å^3^	0.20 × 0.15 × 0.03 mm

### Data collection

**Table:**

Brucker SMART APEX diffractometer	5640 independent reflections
Radiation source: fine-focus sealed tube	3127 reflections with *I* > 2σ(*I*)
Graphite monochromator	*R*_int_ = 0.027
ω scan	θ_max_ = 25.0°, θ_min_ = 1.8°
Absorption correction: multi-scan (*SADABS*; Sheldrick,2008)	*h* = −9→10
*T*_min_ = 0.961, *T*_max_ = 0.994	*k* = −14→14
7929 measured reflections	*l* = −18→15

### Refinement

**Table:**

Refinement on *F*^2^	Primary atom site location: structure-invariant direct methods
Least-squares matrix: full	Secondary atom site location: difference Fourier map
*R*[*F*^2^ > 2σ(*F*^2^)] = 0.058	Hydrogen site location: inferred from neighbouring sites
*wR*(*F*^2^) = 0.155	H atoms treated by a mixture of independent and constrained refinement
*S* = 0.95	*w* = 1/[σ^2^(*F*_o_^2^) + (0.0697*P*)^2^] where *P* = (*F*_o_^2^ + 2*F*_c_^2^)/3
5640 reflections	(Δ/σ)_max_ = 0.001
419 parameters	Δρ_max_ = 0.29 e Å^−^^3^
0 restraints	Δρ_min_ = −0.17 e Å^−^^3^
0 constraints	

### Special details

**Table:**

Geometry. All e.s.d.'s (except the e.s.d. in the dihedral angle between two l.s. planes) are estimated using the full covariance matrix. The cell e.s.d.'s are taken into account individually in the estimation of e.s.d.'s in distances, angles and torsion angles; correlations between e.s.d.'s in cell parameters are only used when they are defined by crystal symmetry. An approximate (isotropic) treatment of cell e.s.d.'s is used for estimating e.s.d.'s involving l.s. planes.
Refinement. Refinement of *F*^2^ against ALL reflections. The weighted *R*-factor *wR* and goodness of fit *S* are based on *F*^2^, conventional *R*-factors *R* are based on *F*, with *F* set to zero for negative *F*^2^. The threshold expression of *F*^2^ > σ(*F*^2^) is used only for calculating *R*-factors(gt) *etc*. and is not relevant to the choice of reflections for refinement. *R*-factors based on *F*^2^ are statistically about twice as large as those based on *F*, and *R*- factors based on ALL data will be even larger.

### Fractional atomic coordinates and isotropic or equivalent isotropic displacement parameters (Å^2^)

**Table:**

	*x*	*y*	*z*	*U*_iso_*/*U*_eq_	
H4N	0.578 (4)	0.812 (3)	0.419 (2)	0.090 (12)*	
S2	1.04941 (11)	0.49993 (7)	1.21135 (6)	0.0688 (3)	
S1	0.71619 (13)	0.85281 (7)	0.21044 (6)	0.0790 (3)	
N8	0.9444 (3)	0.4024 (2)	1.08541 (17)	0.0587 (7)	
N6	0.7262 (3)	0.5924 (2)	1.04273 (16)	0.0592 (7)	
N7	0.8225 (3)	0.5919 (2)	1.10796 (16)	0.0617 (7)	
H7N	0.8092	0.6550	1.1341	0.074*	
N3	0.7612 (3)	0.6926 (2)	0.33190 (16)	0.0667 (8)	
H3N	0.8358	0.6518	0.2990	0.080*	
N2	0.7239 (3)	0.6441 (2)	0.40899 (16)	0.0622 (7)	
C3	0.7517 (4)	0.3395 (3)	0.6503 (2)	0.0591 (8)	
C27	0.9379 (4)	0.4935 (3)	1.13162 (18)	0.0526 (8)	
N4	0.5776 (3)	0.8602 (2)	0.36927 (18)	0.0651 (8)	
C20	0.3119 (3)	0.7492 (3)	0.8192 (2)	0.0540 (8)	
C8	0.7901 (4)	0.4768 (2)	0.50474 (19)	0.0548 (8)	
C25	0.5234 (3)	0.7076 (2)	0.95183 (19)	0.0530 (8)	
C23	0.3245 (4)	0.8396 (3)	0.8656 (2)	0.0591 (8)	
H23A	0.2616	0.9154	0.8530	0.071*	
C28	1.0406 (4)	0.2847 (3)	1.0853 (2)	0.0544 (8)	
C10	0.6811 (4)	0.8030 (3)	0.3086 (2)	0.0600 (8)	
C21	0.4065 (4)	0.6374 (3)	0.8423 (2)	0.0639 (9)	
H21A	0.4001	0.5748	0.8132	0.077*	
N5	0.2121 (3)	0.7702 (2)	0.75317 (19)	0.0726 (8)	
C24	0.4275 (4)	0.8183 (2)	0.9290 (2)	0.0580 (8)	
H24A	0.4340	0.8808	0.9583	0.070*	
C9	0.8135 (4)	0.5414 (3)	0.4282 (2)	0.0614 (9)	
C7	0.8969 (4)	0.3691 (3)	0.5240 (2)	0.0648 (9)	
H7B	0.9836	0.3406	0.4874	0.078*	
C26	0.6322 (4)	0.6915 (3)	1.0191 (2)	0.0576 (8)	
C11	0.4741 (4)	0.9757 (3)	0.37105 (19)	0.0559 (8)	
C22	0.5090 (4)	0.6176 (2)	0.9070 (2)	0.0619 (9)	
H22A	0.5704	0.5418	0.9210	0.074*	
C33	1.0155 (4)	0.2181 (3)	1.0214 (2)	0.0612 (9)	
C6	0.8797 (4)	0.3028 (3)	0.5947 (2)	0.0635 (9)	
H6B	0.9557	0.2315	0.6057	0.076*	
N1	0.7278 (3)	0.2725 (2)	0.71832 (18)	0.0754 (8)	
C16	0.3454 (4)	0.9972 (3)	0.42636 (19)	0.0617 (9)	
C5	0.6640 (4)	0.5151 (3)	0.5611 (2)	0.0727 (10)	
H5A	0.5900	0.5876	0.5505	0.087*	
C4	0.6443 (4)	0.4499 (3)	0.6320 (2)	0.0758 (10)	
H4B	0.5582	0.4793	0.6688	0.091*	
C29	1.1542 (4)	0.2360 (3)	1.1439 (2)	0.0695 (9)	
H29A	1.1705	0.2813	1.1861	0.083*	
C14	0.2744 (4)	1.1999 (3)	0.3839 (2)	0.0814 (11)	
H14A	0.2066	1.2757	0.3886	0.098*	
C30	1.2434 (4)	0.1207 (3)	1.1403 (2)	0.0768 (10)	
H30A	1.3192	0.0875	1.1803	0.092*	
C15	0.2464 (4)	1.1115 (3)	0.4309 (2)	0.0777 (10)	
H15A	0.1585	1.1280	0.4672	0.093*	
C32	1.1080 (5)	0.1035 (3)	1.0189 (2)	0.0777 (11)	
H32A	1.0941	0.0577	0.9763	0.093*	
C31	1.2200 (5)	0.0552 (3)	1.0775 (3)	0.0832 (12)	
H31A	1.2805	−0.0228	1.0746	0.100*	
C13	0.4014 (4)	1.1774 (3)	0.3301 (2)	0.0771 (10)	
H13A	0.4204	1.2373	0.2972	0.092*	
C12	0.5015 (4)	1.0657 (3)	0.3246 (2)	0.0731 (10)	
H12A	0.5897	1.0506	0.2886	0.088*	
C2	0.8405 (4)	0.1598 (3)	0.7369 (2)	0.0862 (12)	
H2B	0.8578	0.1132	0.6872	0.129*	
H2C	0.8027	0.1222	0.7844	0.129*	
H2D	0.9361	0.1689	0.7515	0.129*	
C34	0.8908 (4)	0.2672 (3)	0.9584 (2)	0.0783 (10)	
H34A	0.7927	0.2977	0.9888	0.117*	
H34B	0.8883	0.2071	0.9218	0.117*	
H34C	0.9111	0.3284	0.9240	0.117*	
C19	0.1023 (4)	0.8841 (3)	0.7355 (2)	0.0801 (11)	
H19A	0.0530	0.9171	0.7885	0.120*	
H19B	0.0255	0.8780	0.6982	0.120*	
H19C	0.1552	0.9330	0.7081	0.120*	
C17	0.3151 (5)	0.9008 (3)	0.4802 (2)	0.0911 (12)	
H17A	0.3184	0.8389	0.4439	0.137*	
H17B	0.2147	0.9297	0.5085	0.137*	
H17C	0.3927	0.8722	0.5225	0.137*	
C18	0.2157 (4)	0.6813 (3)	0.6966 (2)	0.0889 (12)	
H18A	0.3104	0.6605	0.6623	0.133*	
H18B	0.1293	0.7093	0.6596	0.133*	
H18C	0.2097	0.6143	0.7297	0.133*	
C1	0.6037 (4)	0.3152 (3)	0.7806 (2)	0.0912 (12)	
H1A	0.5071	0.3430	0.7521	0.137*	
H1B	0.6180	0.3776	0.8101	0.137*	
H1C	0.6029	0.2536	0.8214	0.137*	
H8N	0.864 (3)	0.425 (2)	1.0437 (18)	0.062 (9)*	
H9A	0.903 (3)	0.512 (2)	0.3926 (16)	0.041 (8)*	
H26A	0.635 (4)	0.761 (3)	1.045 (2)	0.082 (11)*	

### Atomic displacement parameters (Å^2^)

**Table:**

	*U*^11^	*U*^22^	*U*^33^	*U*^12^	*U*^13^	*U*^23^
S2	0.0767 (6)	0.0649 (5)	0.0642 (6)	−0.0209 (5)	−0.0089 (4)	−0.0088 (4)
S1	0.1248 (9)	0.0507 (5)	0.0585 (6)	−0.0272 (5)	0.0087 (5)	−0.0021 (4)
N8	0.0627 (19)	0.0517 (16)	0.0603 (17)	−0.0157 (14)	−0.0058 (15)	−0.0080 (14)
N6	0.0618 (18)	0.0560 (17)	0.0585 (17)	−0.0181 (14)	−0.0022 (14)	−0.0015 (13)
N7	0.0688 (19)	0.0515 (16)	0.0644 (17)	−0.0178 (14)	−0.0060 (15)	−0.0105 (13)
N3	0.089 (2)	0.0469 (15)	0.0544 (16)	−0.0118 (14)	0.0088 (15)	0.0007 (13)
N2	0.078 (2)	0.0491 (16)	0.0560 (17)	−0.0172 (15)	−0.0005 (14)	0.0036 (13)
C3	0.074 (2)	0.0452 (18)	0.055 (2)	−0.0161 (17)	−0.0033 (18)	−0.0022 (15)
C27	0.056 (2)	0.0499 (18)	0.0516 (18)	−0.0180 (16)	0.0068 (15)	−0.0011 (15)
N4	0.078 (2)	0.0502 (16)	0.0579 (18)	−0.0105 (15)	0.0004 (15)	0.0052 (14)
C20	0.050 (2)	0.0517 (19)	0.058 (2)	−0.0142 (16)	0.0050 (16)	−0.0031 (15)
C8	0.066 (2)	0.0457 (17)	0.0501 (18)	−0.0146 (16)	−0.0012 (16)	−0.0036 (15)
C25	0.058 (2)	0.0464 (18)	0.0518 (19)	−0.0145 (16)	0.0065 (16)	−0.0031 (14)
C23	0.059 (2)	0.0453 (18)	0.063 (2)	−0.0063 (16)	0.0053 (17)	0.0054 (16)
C28	0.056 (2)	0.0527 (19)	0.0555 (19)	−0.0210 (17)	0.0090 (16)	0.0005 (16)
C10	0.075 (2)	0.0449 (18)	0.061 (2)	−0.0197 (17)	−0.0036 (18)	−0.0047 (16)
C21	0.071 (2)	0.0473 (19)	0.071 (2)	−0.0149 (17)	−0.0082 (19)	−0.0087 (16)
N5	0.070 (2)	0.0604 (18)	0.077 (2)	−0.0055 (15)	−0.0151 (16)	−0.0037 (15)
C24	0.064 (2)	0.0441 (18)	0.063 (2)	−0.0155 (16)	0.0108 (17)	−0.0079 (15)
C9	0.075 (3)	0.051 (2)	0.056 (2)	−0.0177 (19)	0.0027 (19)	−0.0048 (17)
C7	0.070 (2)	0.053 (2)	0.058 (2)	−0.0039 (18)	0.0071 (17)	−0.0050 (16)
C26	0.064 (2)	0.050 (2)	0.060 (2)	−0.0201 (18)	0.0066 (17)	−0.0069 (17)
C11	0.060 (2)	0.0527 (19)	0.0515 (19)	−0.0145 (17)	−0.0028 (16)	−0.0019 (15)
C22	0.062 (2)	0.0412 (17)	0.075 (2)	−0.0084 (16)	0.0012 (18)	−0.0022 (16)
C33	0.074 (2)	0.060 (2)	0.054 (2)	−0.0301 (19)	0.0160 (17)	−0.0107 (16)
C6	0.068 (2)	0.0466 (18)	0.060 (2)	0.0018 (16)	0.0001 (17)	−0.0012 (16)
N1	0.089 (2)	0.0561 (17)	0.0674 (19)	−0.0107 (16)	0.0108 (17)	0.0104 (14)
C16	0.073 (2)	0.064 (2)	0.0496 (19)	−0.0246 (19)	−0.0016 (17)	−0.0051 (16)
C5	0.076 (3)	0.0494 (19)	0.074 (2)	0.0017 (18)	0.001 (2)	0.0050 (18)
C4	0.073 (2)	0.061 (2)	0.072 (2)	0.0006 (19)	0.0183 (19)	0.0071 (18)
C29	0.072 (2)	0.058 (2)	0.076 (2)	−0.0192 (19)	0.001 (2)	−0.0033 (18)
C14	0.084 (3)	0.061 (2)	0.083 (3)	−0.003 (2)	0.008 (2)	−0.006 (2)
C30	0.071 (3)	0.066 (2)	0.083 (3)	−0.011 (2)	0.007 (2)	0.005 (2)
C15	0.070 (3)	0.076 (3)	0.076 (3)	−0.012 (2)	0.0144 (19)	−0.013 (2)
C32	0.101 (3)	0.066 (2)	0.067 (2)	−0.030 (2)	0.022 (2)	−0.0169 (19)
C31	0.101 (3)	0.055 (2)	0.086 (3)	−0.020 (2)	0.033 (3)	−0.009 (2)
C13	0.086 (3)	0.057 (2)	0.080 (3)	−0.016 (2)	0.012 (2)	−0.0020 (18)
C12	0.072 (2)	0.059 (2)	0.078 (2)	−0.0108 (19)	0.0117 (19)	0.0023 (18)
C2	0.119 (3)	0.063 (2)	0.061 (2)	−0.012 (2)	−0.003 (2)	0.0100 (18)
C34	0.101 (3)	0.086 (2)	0.062 (2)	−0.050 (2)	0.003 (2)	−0.0141 (19)
C19	0.066 (2)	0.074 (2)	0.092 (3)	−0.012 (2)	−0.014 (2)	0.010 (2)
C17	0.103 (3)	0.092 (3)	0.083 (3)	−0.041 (2)	0.019 (2)	−0.005 (2)
C18	0.090 (3)	0.086 (3)	0.089 (3)	−0.023 (2)	−0.024 (2)	−0.007 (2)
C1	0.089 (3)	0.095 (3)	0.079 (3)	−0.021 (2)	0.009 (2)	0.015 (2)

### Geometric parameters (Å, º)

**Table:**

S2—C27	1.670 (3)	C22—H22A	0.9300
S1—C10	1.670 (3)	C33—C32	1.374 (4)
N8—C27	1.340 (4)	C33—C34	1.491 (4)
N8—C28	1.413 (4)	C6—H6B	0.9300
N8—H8N	0.97 (3)	N1—C1	1.430 (4)
N6—C26	1.277 (4)	N1—C2	1.443 (4)
N6—N7	1.379 (3)	C16—C15	1.391 (4)
N7—C27	1.357 (4)	C16—C17	1.503 (4)
N7—H7N	0.8600	C5—C4	1.367 (4)
N3—C10	1.343 (3)	C5—H5A	0.9300
N3—N2	1.388 (3)	C4—H4B	0.9300
N3—H3N	0.8600	C29—C30	1.375 (4)
N2—C9	1.279 (4)	C29—H29A	0.9300
C3—N1	1.358 (4)	C14—C13	1.357 (5)
C3—C6	1.382 (4)	C14—C15	1.361 (4)
C3—C4	1.402 (4)	C14—H14A	0.9300
N4—C10	1.347 (4)	C30—C31	1.365 (5)
N4—C11	1.414 (4)	C30—H30A	0.9300
N4—H4N	0.94 (3)	C15—H15A	0.9300
C20—N5	1.369 (4)	C32—C31	1.367 (5)
C20—C21	1.391 (4)	C32—H32A	0.9300
C20—C23	1.391 (4)	C31—H31A	0.9300
C8—C5	1.378 (4)	C13—C12	1.371 (4)
C8—C7	1.381 (4)	C13—H13A	0.9300
C8—C9	1.440 (4)	C12—H12A	0.9300
C25—C22	1.379 (4)	C2—H2B	0.9600
C25—C24	1.383 (4)	C2—H2C	0.9600
C25—C26	1.445 (4)	C2—H2D	0.9600
C23—C24	1.356 (4)	C34—H34A	0.9600
C23—H23A	0.9300	C34—H34B	0.9600
C28—C29	1.378 (4)	C34—H34C	0.9600
C28—C33	1.393 (4)	C19—H19A	0.9600
C21—C22	1.370 (4)	C19—H19B	0.9600
C21—H21A	0.9300	C19—H19C	0.9600
N5—C18	1.426 (4)	C17—H17A	0.9600
N5—C19	1.440 (4)	C17—H17B	0.9600
C24—H24A	0.9300	C17—H17C	0.9600
C9—H9A	0.94 (2)	C18—H18A	0.9600
C7—C6	1.366 (4)	C18—H18B	0.9600
C7—H7B	0.9300	C18—H18C	0.9600
C26—H26A	0.97 (3)	C1—H1A	0.9600
C11—C12	1.370 (4)	C1—H1B	0.9600
C11—C16	1.383 (4)	C1—H1C	0.9600
			
C27—N8—C28	134.1 (3)	C1—N1—C2	116.9 (3)
C27—N8—H8N	110.5 (17)	C11—C16—C15	117.4 (3)
C28—N8—H8N	115.4 (17)	C11—C16—C17	121.2 (3)
C26—N6—N7	116.2 (3)	C15—C16—C17	121.4 (3)
C27—N7—N6	121.1 (3)	C4—C5—C8	122.0 (3)
C27—N7—H7N	119.5	C4—C5—H5A	119.0
N6—N7—H7N	119.5	C8—C5—H5A	119.0
C10—N3—N2	120.1 (3)	C5—C4—C3	121.2 (3)
C10—N3—H3N	120.0	C5—C4—H4B	119.4
N2—N3—H3N	120.0	C3—C4—H4B	119.4
C9—N2—N3	115.3 (3)	C30—C29—C28	120.2 (3)
N1—C3—C6	122.5 (3)	C30—C29—H29A	119.9
N1—C3—C4	121.1 (3)	C28—C29—H29A	119.9
C6—C3—C4	116.5 (3)	C13—C14—C15	119.9 (3)
N8—C27—N7	112.7 (3)	C13—C14—H14A	120.1
N8—C27—S2	129.1 (3)	C15—C14—H14A	120.1
N7—C27—S2	118.2 (2)	C31—C30—C29	119.6 (4)
C10—N4—C11	131.7 (3)	C31—C30—H30A	120.2
C10—N4—H4N	111 (2)	C29—C30—H30A	120.2
C11—N4—H4N	117 (2)	C14—C15—C16	121.9 (3)
N5—C20—C21	121.8 (3)	C14—C15—H15A	119.0
N5—C20—C23	121.2 (3)	C16—C15—H15A	119.0
C21—C20—C23	117.0 (3)	C31—C32—C33	121.6 (4)
C5—C8—C7	116.5 (3)	C31—C32—H32A	119.2
C5—C8—C9	123.9 (3)	C33—C32—H32A	119.2
C7—C8—C9	119.6 (3)	C30—C31—C32	120.4 (3)
C22—C25—C24	116.6 (3)	C30—C31—H31A	119.8
C22—C25—C26	123.6 (3)	C32—C31—H31A	119.8
C24—C25—C26	119.7 (3)	C14—C13—C12	119.5 (3)
C24—C23—C20	120.8 (3)	C14—C13—H13A	120.3
C24—C23—H23A	119.6	C12—C13—H13A	120.3
C20—C23—H23A	119.6	C11—C12—C13	121.2 (3)
C29—C28—C33	120.5 (3)	C11—C12—H12A	119.4
C29—C28—N8	123.2 (3)	C13—C12—H12A	119.4
C33—C28—N8	116.3 (3)	N1—C2—H2B	109.5
N3—C10—N4	113.9 (3)	N1—C2—H2C	109.5
N3—C10—S1	118.4 (2)	H2B—C2—H2C	109.5
N4—C10—S1	127.7 (2)	N1—C2—H2D	109.5
C22—C21—C20	121.4 (3)	H2B—C2—H2D	109.5
C22—C21—H21A	119.3	H2C—C2—H2D	109.5
C20—C21—H21A	119.3	C33—C34—H34A	109.5
C20—N5—C18	121.6 (3)	C33—C34—H34B	109.5
C20—N5—C19	121.7 (3)	H34A—C34—H34B	109.5
C18—N5—C19	116.7 (3)	C33—C34—H34C	109.5
C23—C24—C25	122.7 (3)	H34A—C34—H34C	109.5
C23—C24—H24A	118.7	H34B—C34—H34C	109.5
C25—C24—H24A	118.7	N5—C19—H19A	109.5
N2—C9—C8	122.8 (3)	N5—C19—H19B	109.5
N2—C9—H9A	116.4 (15)	H19A—C19—H19B	109.5
C8—C9—H9A	120.4 (15)	N5—C19—H19C	109.5
C6—C7—C8	122.3 (3)	H19A—C19—H19C	109.5
C6—C7—H7B	118.8	H19B—C19—H19C	109.5
C8—C7—H7B	118.8	C16—C17—H17A	109.5
N6—C26—C25	123.4 (3)	C16—C17—H17B	109.5
N6—C26—H26A	119.9 (19)	H17A—C17—H17B	109.5
C25—C26—H26A	116.6 (18)	C16—C17—H17C	109.5
C12—C11—C16	120.0 (3)	H17A—C17—H17C	109.5
C12—C11—N4	122.7 (3)	H17B—C17—H17C	109.5
C16—C11—N4	117.2 (3)	N5—C18—H18A	109.5
C21—C22—C25	121.5 (3)	N5—C18—H18B	109.5
C21—C22—H22A	119.2	H18A—C18—H18B	109.5
C25—C22—H22A	119.2	N5—C18—H18C	109.5
C32—C33—C28	117.8 (3)	H18A—C18—H18C	109.5
C32—C33—C34	120.3 (3)	H18B—C18—H18C	109.5
C28—C33—C34	121.9 (3)	N1—C1—H1A	109.5
C7—C6—C3	121.4 (3)	N1—C1—H1B	109.5
C7—C6—H6B	119.3	H1A—C1—H1B	109.5
C3—C6—H6B	119.3	N1—C1—H1C	109.5
C3—N1—C1	122.2 (3)	H1A—C1—H1C	109.5
C3—N1—C2	120.3 (3)	H1B—C1—H1C	109.5

### Hydrogen-bond geometry (Å, º)

**Table:**

*D*—H···*A*	*D*—H	H···*A*	*D*···*A*	*D*—H···*A*
N4—H4*N*···N2	0.95 (3)	2.05 (3)	2.587 (3)	114 (3)
C12—H12*A*···S1	0.93	2.64	3.239 (4)	123
C29—H29*A*···S2	0.93	2.57	3.257 (4)	131
N7—H7*N*···S1^i^	0.86	2.61	3.458 (3)	170
N3—H3*N*···S2^ii^	0.86	2.59	3.439 (3)	171
N8—H8*N*···N6	0.97 (3)	2.01 (3)	2.587 (4)	116 (2)

Symmetry codes: (i) *x*, *y*, *z*+1; (ii) *x*, *y*, *z*−1.
