# 1-[4-(Di­methyl­amino)­benzyl­idene]-4-*o*-tolyl­thio­semicarbazide. Corrigendum

**DOI:** 10.1107/S1600536813016218

**Published:** 2013-06-15

**Authors:** Rui-Yun Huang, Qin-Juan Xu, Li-Rong Lin

**Affiliations:** a Department of Chemistry, College of Chemistry and Chemical Engineering, Xiamen University, Xiamen 361005, People’s Republic of China

## Abstract

The correspondence address in the paper by Huang *et al.* [*Acta Cryst.* (2013), E**69**, o906–o907] is corrected.

## Abstract 2

Corrigendum to *Acta Cryst.* (2013), E**69**, o906–o907.

In the paper by Huang *et al.* (2013) ▶, the correspondence address was incomplete. The correct full address is given above.
